# Differential associations between job crafting dimensions and work engagement: a meta-analytic review based on the JD–R framework

**DOI:** 10.3389/fpsyg.2026.1856109

**Published:** 2026-10-23

**Authors:** Zhongrui Qiao, Anton F. Gasimov

**Affiliations:** 1 Department of Labour and Engineering Psychology, Faculty of Psychology, Lomonosov Moscow State University, Moscow, Russia; 2 Department of Social Psychology, Faculty of Psychology, Lomonosov Moscow State University, Moscow, Russia

**Keywords:** challenging job demands, hindering job demands, job crafting, Job Demands–Resources (JD–R) framework, meta-analysis, social job resources, structural job resources, work engagement

## Abstract

Work engagement is a positive work-related state characterized by vigor, dedication, and absorption. Within the Job Demands–Resources (JD–R) framework, job crafting has commonly been conceptualized as a set of proactive behaviors through which employees modify job resources and demands to better align work with their abilities and motivations. Although previous meta-analyses have linked job crafting to work engagement and provided initial evidence of dimension-specific patterns, the extent to which these patterns hold across a broader and more recent evidence base remains insufficiently clarified. To address this issue, the present study conducted an updated random-effects meta-analysis of the associations between four job crafting dimensions conceptualized within the JD–R framework and work engagement. A systematic search of Web of Science, ScienceDirect, PsycINFO, and PubMed identified 20 eligible studies, and five additional studies were identified through reviewer recommendation during peer review, yielding 25 studies for narrative synthesis. Of these, 15 entered the quantitative synthesis. The findings indicated a differentiated pattern across dimensions. Increasing structural job resources showed the strongest positive pooled association with work engagement (*r* = 0.491, 95% CI [0.340, 0.617]). Increasing social job resources was also positively associated with work engagement (*r* = 0.345, 95% CI [0.226, 0.454]), and increasing challenging job demands likewise showed a significant positive association (*r* = 0.422, 95% CI [0.299, 0.532]). By contrast, decreasing hindering job demands showed a near-null and non-significant association (*r* = 0.032, 95% CI [−0.087, 0.149]). Sensitivity analyses generally supported the primary pattern of findings, although the association for decreasing hindering job demands showed greater sensitivity to study design, and substantial between-study heterogeneity remained across the syntheses. Overall, the results suggest that JD–R-based job crafting dimensions should not be treated as functionally equivalent when work engagement is the focal outcome. The paper concludes by discussing the theoretical implications of a differentiated view of job crafting, the practical relevance of dimension-specific job crafting for organizations, and directions for future research.

## 1 Introduction

Modern work systems are increasingly characterized by technological complexity, dynamic task demands, and intensified performance pressures (Parker and Grote, 2022). Within such environments, employees are required not only to execute predefined tasks but also to continuously regulate their effort, allocate cognitive resources, and adapt to fluctuating work demands. Understanding how employees maintain sustained motivation and effective functioning under such conditions is therefore important for explaining adaptive functioning in contemporary work environments.

One construct that captures adaptive functioning in demanding work environments is work engagement. Work engagement refers to a positive, work-related state characterized by vigor, dedication, and absorption, reflecting the degree to which employees mobilize energetic, cognitive, and motivational resources while performing their work tasks (Bakker et al., 2014). Within the Job Demands–Resources (JD–R) framework, engagement represents a key indicator of motivational functioning. The model proposes that job resources are linked to engagement through motivational processes, whereas excessive job demands are associated with strain-related impairment processes (Demerouti et al., 2001; Bakker and Demerouti, 2008). Consistent with this perspective, empirical evidence indicates that engaged employees demonstrate higher levels of proactive performance and organizational citizenship behaviors (Lee and Jo, 2023).

Traditional job design approaches—particularly those derived from the Job Characteristics Model—have largely emphasized top-down managerial control over task structure and resource allocation. In contrast, contemporary work contexts increasingly provide employees with greater autonomy to shape their own work roles. This shift has drawn scholarly attention to job crafting, defined as self-initiated adjustments through which employees modify aspects of their tasks, relationships, or work boundaries to better align job demands and resources with their abilities and motivations. Longitudinal research has shown that job crafting can influence subsequent job demands, job resources, and well-being (Tims et al., 2013). Evidence further suggests that increasing structural resources, seeking social resources, and pursuing challenging demands are positively associated with work engagement and subsequent performance outcomes.

The JD–R framework provides a useful theoretical lens for understanding the mechanisms underlying job crafting behaviors. According to this model, all jobs contain job demands, which require sustained effort and may incur psychological costs, and job resources, which facilitate goal attainment, support learning, and buffer the negative impact of demands. Importantly, the JD–R framework distinguishes between two psychological processes: a motivational pathway, through which job resources foster engagement, and a health-impairment pathway, through which excessive demands contribute to strain and burnout (Schaufeli, 2017).

Building on this framework, job crafting has commonly been conceptualized as comprising four behavioral dimensions: increasing structural job resources, increasing social job resources, increasing challenging job demands, and decreasing hindering job demands. Resource-expanding behaviors, such as acquiring new skills or seeking feedback, are theoretically aligned with greater access to job resources and with the motivational pathway. Similarly, increasing challenging job demands may be associated with opportunities for learning, mastery, and greater task involvement. By comparison, decreasing hindering job demands is more closely aligned with strain regulation and resource conservation. Consistent with this distinction, research grounded in the JD–R framework has linked demanding work conditions, such as physical strain and secondary trauma, with higher levels of burnout, whereas job resources have been associated with lower levels of burnout (Patel and Bartholomew, 2021).

Conceptually, these different crafting strategies may operate through partially distinct psychological mechanisms. Resource- and challenge-expanding behaviors are generally more approach- or promotion-oriented, whereas decreasing hindering job demands is more closely aligned with prevention, demand regulation, and resource conservation (Lichtenthaler and Fischbach, 2019). Consequently, job crafting dimensions may show differential associations with work engagement. Empirical research provides support for this possibility. Diary evidence indicates that day-level seeking challenges is positively associated with day-level work engagement, whereas reducing demands has shown negative associations with engagement and seeking resources has not shown a consistent association with engagement at the within-person level (Petrou et al., 2012). This pattern suggests that different crafting behaviors may be differentially related to work engagement even within the same theoretical framework.

Nevertheless, a substantial portion of the literature has operationalized job crafting using overall or higher-order scores that combine distinct crafting dimensions. Although such aggregation enhances parsimony, it may implicitly treat different crafting strategies as functionally interchangeable. This approach can obscure theoretically meaningful differences between resource- and challenge-expanding behaviors and demand-reducing forms of crafting.

Recent empirical findings further suggest that different job crafting dimensions may show differential associations with organizational outcomes. For instance, Szőts-Kováts and Kiss (2023) reported that different role-based job crafting strategies exhibited distinct relationships with employees’ readiness to change, with some dimensions showing significant associations whereas others showed null associations. These findings suggest that specific job crafting behaviors may not be functionally interchangeable in their relationships with employee outcomes.

Previous meta-analytic research has provided important evidence concerning the relationship between job crafting and work engagement. Rudolph et al. (2017) synthesized associations between job crafting, individual differences, job characteristics, and multiple work outcomes, including work engagement. Lichtenthaler and Fischbach (2019) distinguished promotion- and prevention-focused forms of job crafting and examined their associations with work-related outcomes. More specifically, Frederick and VanderWeele (2020) focused on repeated-measures designs and reported meta-analytic estimates for overall job crafting as well as its structural, social, challenging, and hindering components in relation to subsequent work engagement. Together, these studies indicate an overall positive relationship between job crafting and work engagement while also suggesting meaningful variation across forms of job crafting.

However, several issues remain open. Earlier syntheses differed substantially in their conceptual scope and design restrictions: some examined job crafting in relation to a broad range of work outcomes or grouped crafting behaviors into higher-order categories, whereas Frederick and VanderWeele (2020) deliberately restricted their synthesis to repeated-measures designs. The literature has also continued to expand, with considerable variation in study design and in the operationalization of the four job crafting dimensions. An updated synthesis that focuses specifically on work engagement, estimates dimension-specific associations within a common JD–R framework, and evaluates the robustness of these associations across study designs and operationalizations may therefore provide a more current and methodologically differentiated account of the evidence.

Building on this earlier meta-analytic literature, the present study provides an updated synthesis of the associations between specific job crafting dimensions and work engagement. Separate pooled associations are estimated for increasing structural job resources, increasing social job resources, increasing challenging job demands, and decreasing hindering job demands. The quality of the evidence is evaluated through a design-specific risk-of-bias assessment, while the robustness of the pooled associations is examined using leave-one-out analyses and sensitivity analyses based on study design and construct operationalization. This approach allows the review to clarify both the pattern of dimension-specific associations and the extent to which that pattern remains stable when studies with weaker temporal designs or less directly aligned operationalizations are excluded.

## 2 Methods

### 2.1 Literature search

A systematic literature search was conducted to identify empirical studies examining the relationship between job crafting and work engagement. The search was performed across four major academic databases: Web of Science, ScienceDirect, PsycINFO, and PubMed. The search strategy was structured around the broad core concepts of “job crafting” and “work engagement.” To maximize retrieval sensitivity, dimension-specific job crafting terms were included within the same search block rather than used as mandatory eligibility conditions. The Boolean search expression was as follows: (“job crafting” OR “increasing structural job resources” OR “increasing social job resources” OR “increasing challenging job demands” OR “decreasing hindering job demands”) AND (“work engagement”). Because the broad term “job crafting” was retained in the OR block, records were not required to mention a specific job crafting dimension at the search stage; dimension-specific relevance was evaluated subsequently during study screening.

Searches were conducted primarily within the title, abstract, and keyword fields, where applicable. Only peer-reviewed articles published in English were included. The search was restricted to studies published from 2008 onward. The lower boundary was selected to provide a conservative lead-in period preceding the transition from the broader task-, relational-, and cognitive-crafting tradition introduced by Wrzesniewski and Dutton (2001) to the resource- and demand-based conceptualization of job crafting within the JD–R framework. Job crafting was formally reframed in JD–R terms by Tims and Bakker (2010), followed by the development and validation of the four-dimensional Job Crafting Scale by Tims et al. (2012). Beginning the search in 2008 was therefore intended to reduce the likelihood of excluding early empirical work emerging around this conceptual transition. The same lower publication boundary was also used by Frederick and VanderWeele (2020) in their earlier meta-analysis of job crafting and work engagement. The search was conducted in March 2026, and all retrieved records were exported to reference management software for duplicate removal and screening. Study screening and eligibility assessment were performed by the first author according to predefined inclusion and exclusion criteria. The study selection procedure followed the PRISMA 2020 guidelines (Page et al., 2021).

### 2.2 Eligibility criteria and review focus

To ensure conceptual clarity and methodological comparability across studies, explicit eligibility criteria were established prior to the screening process. The present meta-analysis focused on empirical research examining the association between job crafting and work engagement within organizational settings. Studies were considered eligible if they investigated the relationship between overall job crafting or its specific dimensions—such as increasing structural job resources, increasing social job resources, increasing challenging job demands, or decreasing hindering job demands—and work engagement. In addition, studies were required to assess work engagement using validated psychometric instruments, most commonly the Utrecht Work Engagement Scale (UWES; Schaufeli et al., 2006) or its abbreviated versions. To enable quantitative synthesis, eligible studies had to report sufficient statistical information allowing the computation or transformation of effect sizes, including Pearson correlations, standardized regression coefficients, or other convertible statistics. Furthermore, studies were required to involve employees working in real organizational contexts, and to adopt an empirical quantitative design suitable for meta-analytic integration, including cross-sectional, longitudinal, diary, or experimental methodologies.

Studies were excluded if they did not directly examine job crafting or its specific dimensions, did not measure work engagement as an outcome variable, or failed to provide sufficient statistical information necessary for calculating effect sizes. In addition, studies based on non-employee samples (e.g., student populations) were excluded in order to maintain ecological validity in relation to workplace contexts. Furthermore, theoretical papers, conceptual articles, literature reviews, qualitative studies, conference abstracts, dissertations, and other non-peer-reviewed publications were excluded to ensure the methodological rigor and comparability of the synthesized evidence. Collectively, these criteria ensured that the included studies were conceptually aligned with the research focus and provided statistically comparable estimates suitable for meta-analytic integration.

### 2.3 Literature search results

We sought to identify empirical studies examining the association between job crafting and work engagement in organizational settings. To this end, we conducted a systematic literature search across four databases: Web of Science, ScienceDirect, PsycINFO, and PubMed (see Figure 1 for the PRISMA flow diagram). The initial search yielded 268 records, comprising 125 records from Web of Science, 126 from ScienceDirect, 15 from PsycINFO, and two from PubMed. After the removal of 20 duplicate records, 248 unique articles remained for screening.

**FIGURE 1:**
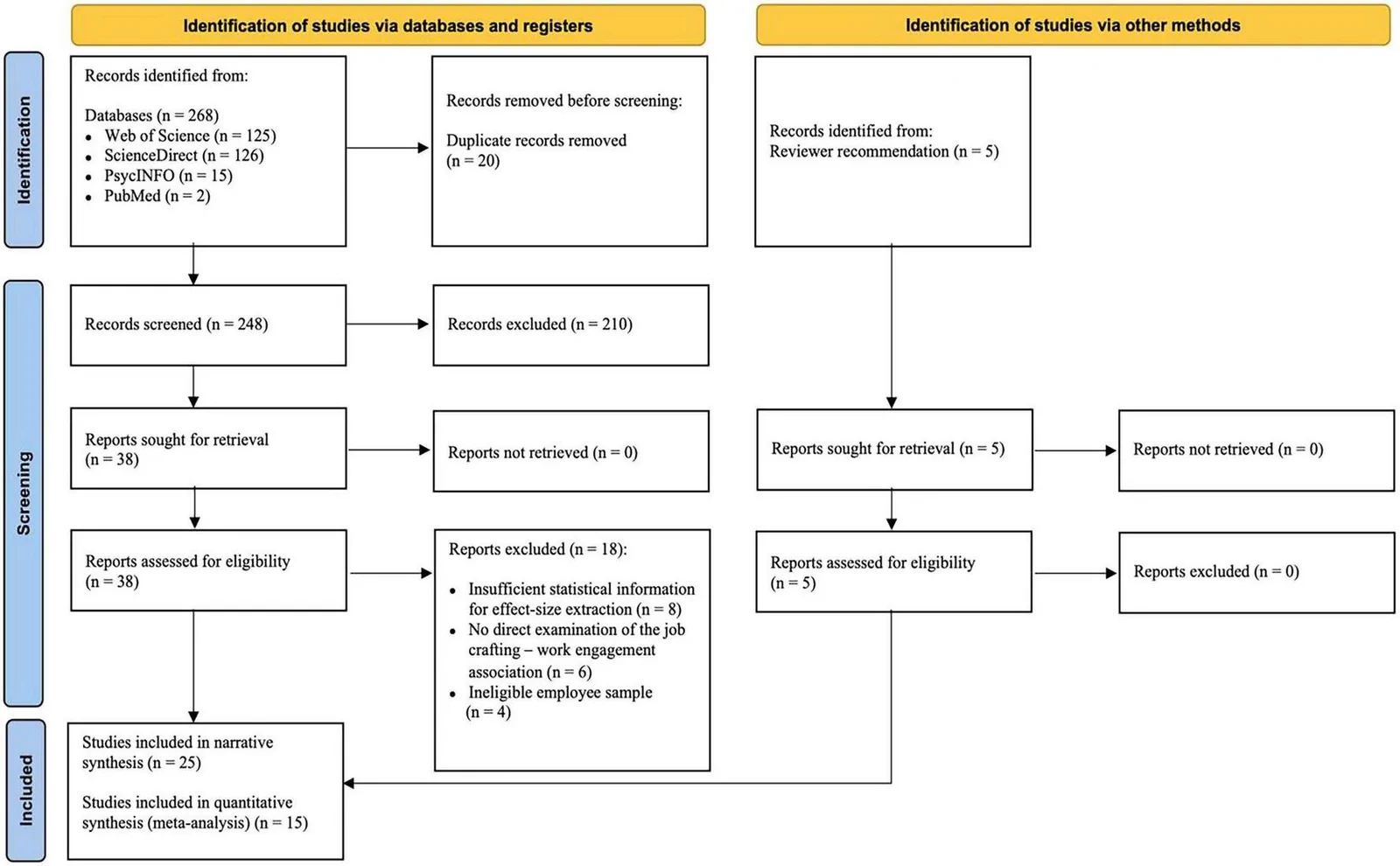
PRISMA 2020 flow diagram of the literature selection process. Adapted from the PRISMA 2020 flow diagram (Page et al., 2021), licensed under CC BY 4.0.

The screening stage involved examining titles, abstracts, and keywords to determine whether the retrieved studies addressed the relationship between job crafting and work engagement and met the predefined inclusion criteria. At this stage, studies were excluded if they did not assess work engagement, did not focus on job crafting, relied on non-employee samples, or were theoretical or review papers rather than empirical investigations. Following this initial screening, 38 articles were retained for full-text assessment. Dimension-specific relevance and other eligibility requirements were therefore applied during screening rather than imposed as mandatory search conditions.

We assessed 38 full-text reports for eligibility, of which 18 were excluded for prespecified reasons: insufficient statistical information to derive a usable job crafting–work engagement effect estimate (*n* = 8), no direct examination of the job crafting–work engagement association (*n* = 6), or an ineligible employee sample (*n* = 4). This left 20 studies from the database search for narrative synthesis. During the second round of peer review, five additional studies were identified through reviewer recommendation and evaluated using the same predefined eligibility criteria. All five met the review-level inclusion criteria, bringing the narrative synthesis to 25 studies, and four also provided sufficiently comparable and extractable estimates for inclusion in the quantitative synthesis.

Across the 25 studies retained for narrative synthesis, job crafting was assessed primarily using measures derived from the Job Crafting Scale (Tims et al., 2012) or conceptually related adaptations. Several studies used shortened or modified versions in which structural and social resources were combined into a broader resource-seeking dimension or multiple crafting components were collapsed into fewer factors. Others adopted role-based operationalizations, including task, relational, and cognitive crafting. Work engagement was assessed using validated instruments, most commonly the Utrecht Work Engagement Scale (UWES; Schaufeli et al., 2006), capturing vigor, dedication, and absorption. Study designs included cross-sectional surveys, longitudinal studies, diary designs, and intervention-based research.

Of the 25 studies retained for narrative synthesis, 15 contributed to the quantitative synthesis. The remaining 10 were not pooled because their reported estimates were not sufficiently commensurate with the dimension-specific analytic framework used in the meta-analysis. Two studies (Hakanen et al., 2018; Huyghebaert-Zouaghi et al., 2021) modeled work engagement, or broader wellbeing indicators, as antecedents rather than outcomes of job crafting. Kuijpers et al. (2020), Yang et al. (2017) used conceptually relevant but operationally different forms of job crafting that could not be mapped directly onto the JD–R-based dimensions synthesized here. Radstaak and Hennes (2017) examined job crafting within an LMX–engagement mediation framework but did not provide a dimension-specific estimate sufficiently comparable with the primary pooled models. Baghdadi et al. (2021) reported only highly aggregated evidence and did not provide extractable dimension-specific statistics. van Wingerden and Poell (2017) examined a sequential model linking perceived opportunities to craft, job crafting, and work engagement and did not assess decreasing hindering job demands. Alkhraishi and Yesiltas (2024), Lee et al. (2017) operationalized job crafting in role-based terms, including task, cognitive, and relational crafting, rather than in resource- and demand-based dimensions. Vieira Dos Santos et al. (2023) was retained for narrative synthesis because it examined the four relevant job crafting dimensions in relation to work engagement. However, the study reported separate associations with vigor, dedication, and absorption rather than a single directly extractable association with overall work engagement. Because these facet-specific estimates were derived from the same sample and were therefore statistically dependent, they were not combined *post hoc* into a single effect estimate. Taken together, these studies were retained in the narrative synthesis but excluded from pooled estimation to preserve conceptual and statistical comparability across the quantitative analyses.

In total, 15 studies were retained for the quantitative synthesis (see Table 1). These studies were methodologically heterogeneous, comprising three-wave longitudinal studies, two-wave longitudinal studies, a 5-day diary study, cross-sectional surveys, a dyadic survey study, a quasi-experimental intervention study, and related repeated-measures designs. Despite this variation in design, all retained studies examined the association between job crafting and work engagement in employee samples and provided sufficiently comparable statistical information for effect size extraction.

**TABLE 1:** Characteristics of studies included in the meta-analysis.

References	Country	Sample size	Design	Job crafting operationalization	Original dimension labels reported	Work engagement measure	Effect-size source
Bakker et al., 2016	Poland, Romania, Lithuania, and Netherlands	206	Dyadic survey	JD-R-based job crafting scale following Tims et al. (2012)	Increasing structural job resources; increasing social job resources; increasing challenging job demands; decreasing hindering job demands	Work engagement operationalized through vigor, dedication, and absorption	Pearson correlations derived from dyadic SEM and APIM estimates
Tims et al., 2015	Netherlands	288	Three-wave longitudinal (1-month lags)	Full JD–R-based Job Crafting Scale	Crafting job resources; crafting challenging job demands; crafting hindering job demands	Work engagement	Standardized SEM coefficients
Petrou et al., 2012	Netherlands	95	Five-day diary study	Daily job crafting measure developed within a JD-R framework	Seeking resources; seeking challenges; reducing demands	Daily work engagement	Pearson correlations
Dubbelt et al., 2019, Study 1	Netherlands	111	Longitudinal survey	JD–R-related job crafting operationalization	Seeking resources; seeking challenges; decreasing demands	Work engagement	Pearson correlations
Dubbelt et al., 2019, Study 2	Netherlands	119	Quasi-experimental intervention	JD–R-related job crafting operationalization	Seeking resources; seeking challenges; decreasing demands	Work engagement	Standardized multilevel coefficients
Tims et al., 2013	Netherlands	288	Three-wave longitudinal study (1-month intervals)	Full JD–R-based Job Crafting Scale	Crafting structural job resources; crafting social job resources; crafting challenging job demands; crafting hindering job demands	Work engagement	Standardized SEM coefficients
Bakker et al., 2012	Netherlands	190	Cross-sectional dyadic survey	JD–R-based job crafting scale	Increasing structural job resources; increasing social job resources; increasing job challenges	Work engagement operationalized through vigor, dedication, and absorption	Standardized SEM coefficients
Moreira et al., 2022	Portugal	453	Cross-sectional survey	JD–R-based job crafting measure	Increasing structural job resources; increasing social job resources; increasing challenging job demands; decreasing hindering job demands	UWES-9	Pearson correlations
De Beer et al., 2016, mining sample	South Africa	260	Cross-sectional survey	Job Crafting Scale with partial three-factor adjustment due to discriminant validity concerns	Integrated crafting of structural resources and challenging job demands; increasing social job resources	Work engagement	Standardized SEM coefficients
De Beer et al., 2016, manufacturing sample	South Africa	210	Cross-sectional survey	Job Crafting Scale with partial three-factor adjustment due to discriminant validity concerns	Integrated crafting of structural resources and challenging job demands; increasing social job resources	Work engagement	Standardized SEM coefficients
Petrou et al., 2018	Netherlands	368	Three-wave longitudinal study in an organizational change context	JD–R-related job crafting measure	Seeking resources; reducing demands	Work engagement	Latent change score coefficients
Muttar et al., 2024	Bahrain	276	Cross-sectional survey	21-item job crafting scale adapted from Bakker et al. (2012) within the JD–R framework	Increasing structural job resources; decreasing hindering job demands; increasing social job resources; increasing challenging job demands	17-item scale derived from Schaufeli and Bakker (2003)	Standardized regression coefficients
Sakuraya et al., 2017	Japan	894	Cross-sectional internet survey	Japanese version of the Job Crafting Questionnaire based on the JD–R framework	Increasing structural job resources; increasing social job resources; increasing challenging job demands; decreasing hindering job demands	Japanese version of the Utrecht Work Engagement Scale (UWES-9)	Hierarchical multiple regression analysis with demographics controlled in Step 1 and job crafting dimensions entered in Step 2
Moulik and Giri, 2024	India	310	Cross-sectional survey	Job Crafting Scale based on Tims et al. (2012)	Seeking structural job resources; seeking social job resources; seeking challenging job demands	Work engagement measure based on Schaufeli et al. (2002)	Pearson correlations
Ariani, 2023	Indonesia	1,032	Cross-sectional survey	11-item job crafting measure adapted from Petrou et al. (2012)	Seeking job resources; seeking job challenges; reducing job demands	17-item employee engagement measure based on Schaufeli and Bakker (2003), modeled as an overall construct	Pearson correlations
Chen et al., 2024	China	733	Cross-sectional survey	Modified 12-item job crafting scale based on Tims et al. (2012), Petrou et al. (2012)	Seeking resources; seeking challenges; reducing demands	UWES-9	Pearson correlations
Wang et al., 2024	China	308	Three-wave longitudinal study (2-week intervals)	13-item job crafting scale developed by Petrou et al. (2012)	Seeking resources; seeking challenges; reducing demands	17-item work engagement scale based on Schaufeli et al. (2002)	Pearson correlations

Studies retained for narrative synthesis only are discussed separately in the main text. Studies are reported at the sample level rather than the article level to reflect the analytic structure of the meta-analysis. When a single article contributed multiple analytically distinct samples or sub-studies, these were presented in separate rows. For studies using the three-dimensional Petrou et al. (2012) operationalization, seeking challenges and reducing demands were mapped to increasing challenging job demands and decreasing hindering job demands, respectively. The broader seeking resources construct was not assigned to either the structural or social resource dimension because it combines multiple forms of resource seeking. APIM, Actor–Partner Interdependence Model; JD–R, Job Demands–Resources; SEM, structural equation modeling; UWES, Utrecht Work Engagement Scale.

Job crafting was operationalized primarily within the JD–R framework proposed by Tims et al. (2012) or closely related adaptations. Most studies assessed increasing structural resources, increasing social resources, increasing challenging demands, and decreasing hindering demands. Some, however, used shortened or conceptually adjacent versions. In particular, several adopted the three-dimensional format derived from Petrou et al. (2012), which combines structural and social resource seeking into a broader seeking resources factor alongside seeking challenges and reducing demands. Other studies used broader distinctions, including approach versus avoidance crafting and expansive versus reduction-oriented crafting, and were retained only when these operationalizations were judged sufficiently consistent with the JD–R distinction between resource- and challenge-expanding crafting and demand-reducing crafting.

Work engagement was assessed using validated instruments in all included studies, most commonly the Utrecht Work Engagement Scale (UWES) or closely related operationalizations based on vigor, dedication, and absorption (Schaufeli et al., 2002). Although the measurement formats and analytic approaches varied across studies, the retained estimates were considered sufficiently commensurate with the analytic focus of the present review, namely the differential associations between specific job crafting dimensions and work engagement. For this reason, studies with clearly non-comparable analytic direction, highly aggregated reporting, or construct operationalizations that fell outside the JD–R-based dimension framework were discussed narratively but not entered into the pooled quantitative synthesis.

### 2.4 Data extraction and effect-size transformation

For each study included in the quantitative synthesis, we extracted the sample size, study design, job crafting operationalization, work engagement measure, and the statistical information required to estimate the association between job crafting and work engagement. Because the present meta-analysis focused on the differential relationships between specific JD–R-based job crafting dimensions and work engagement, effect sizes were extracted separately for increasing structural job resources, increasing social job resources, increasing challenging job demands, and decreasing hindering job demands whenever such information was available. When studies used conceptually related labels, such as seeking challenges or reducing demands, these were coded into the closest corresponding JD–R dimension only when the underlying construct meaning was judged to be sufficiently aligned with the focal dimension.

A structured effect-size selection hierarchy was applied in order to maximize comparability across studies. When zero-order correlations between a focal job crafting dimension and work engagement were reported, Pearson’s r was extracted directly. When zero-order correlations were unavailable, standardized coefficients from SEM, regression, multilevel, or latent change models were retained only when they represented the association between a focal job crafting dimension and work engagement in a direction consistent with the review question and could be interpreted as standardized association estimates. When multiple coefficients were reported for the same conceptual dimension within a single sample, preference was given to the estimate judged to be most directly aligned with the focal predictor–outcome association and least affected by additional model complexity or aggregation (Scammacca et al., 2014).

When studies reported unstandardized regression coefficients and the corresponding standard deviations of the predictor and outcome were available, the coefficients were standardized as follows:


$$\beta =b\,\left(\frac{S\,D\,x}{S\,D\,y}\right)$$

where *b* is the unstandardized coefficient, *SD*_*x*_ is the standard deviation of the focal job crafting variable, and *SD*_*y*_ is the standard deviation of work engagement. In longitudinal studies, the standard deviation of the predictor at the earlier time point and the standard deviation of work engagement at the later time point were used. All effect sizes were then translated into a common correlational metric (Frederick and VanderWeele, 2020). Where necessary, effects expressed in standardized mean-difference form could be converted to r using the following transformation:


$$r=\frac{d}{\sqrt{d^{2}+4}}$$

To stabilize sampling variance, all correlation coefficients were transformed to Fisher’s *z* prior to meta-analysis:


$$z=\frac{1}{2}\,\ln\left(\frac{1+r}{1-r}\right)$$

with sampling variance estimated as:


$$V\,z=\frac{1}{N-3}$$

where *N* denotes the sample size. Pooled estimates were subsequently back-transformed to Pearson’s r for interpretation.

Because several retained studies reported standardized coefficients rather than zero-order correlations, the resulting effect sizes were treated as standardized approximations of association strength rather than as strictly identical statistical quantities (Nieminen, 2022; Peterson and Brown, 2005). For this reason, the primary synthesis was complemented by sensitivity analyses designed to evaluate whether the substantive conclusions were materially altered when broader or less directly aligned operationalizations were excluded.

These transformations were implemented at the level of the individual study estimate prior to model fitting. When multiple coefficients from the same sample referred to different job crafting dimensions, they were retained separately for the relevant dimension-specific syntheses. When multiple coefficients represented the same conceptual dimension within one sample, they were combined before analysis so that each sample contributed no more than one effect size per dimension. This rule was adopted to preserve conceptual differentiation across dimensions while minimizing statistical dependency within each synthesis. Table 2 provides a study-level overview of these extraction and standardization decisions, including the original reported metrics, the corresponding standardized effect sizes, Fisher’s *z* transformations, and inverse-variance weights used in the primary meta-analytic models.

**TABLE 2:** Extraction and standardization of effect sizes across studies included in the primary meta-analytic synthesis.

References	Sample size (*N*)	Job crafting dimension	Effect size metric reported	Standardized effect size (*r*)	Fisher’s *z* transfor mation	Inverse-variance weight (*N*−3)
Bakker et al., 2016	206	Increasing structural job resources	Pearson r = 0.57	0.57	0.648	203
	206	Increasing social job resources	Pearson r = 0.26	0.26	0.266	203
	206	Increasing challenging job demands	Pearson r = 0.49	0.49	0.536	203
	206	Decreasing hindering job demands	Pearson r = −0.07	−0.07	−0.070	203
Tims et al., 2015	288	Crafting job resources and challenging job demands	SEM β = 0.20	0.20	0.203	285
	288	Crafting hindering job demands	SEM β = −0.06	−0.06	−0.060	285
Petrou et al., 2012	95	Seeking resources	Pearson r = 0.31	0.31	0.321	92
	95	Seeking challenges	Pearson r = 0.40	0.40	0.424	92
	95	Reducing demands	Pearson r = 0.03	0.03	0.030	92
Dubbelt et al., 2019, Study 1	111	Seeking challenges	Pearson r = 0.04	0.04	0.040	108
	111	Seeking resources	Pearson r = 0.27	0.27	0.277	108
	111	Decreasing demands	Pearson r = −0.14	−0.14	−0.141	108
Dubbelt et al., 2019, Study 2	119	Seeking resources	Multilevel β = 0.78	0.78	1.045	116
	119	Seeking challenges	Multilevel β = 0.12	0.12	0.121	116
	119	Decreasing demands	Multilevel β = −0.17	−0.17	−0.172	116
Tims et al., 2013	288	Crafting structural job resources	SEM β = 0.17	0.17	0.172	285
	288	Crafting social job resources	SEM β = 0.19	0.19	0.192	285
	288	Crafting challenging job demands	SEM β = 0.10	0.10	0.100	285
	288	Crafting hindering job demands	SEM β = 0.05	0.05	0.050	285
Bakker et al., 2012	190	Increasing structural job resources	SEM β = 0.69	0.69	0.848	187
	190	Increasing social job resources	SEM β = 0.47	0.47	0.510	187
	190	Increasing job challenges	SEM β = 0.65	0.65	0.775	187
Moreira et al., 2022	453	Increasing structural job resources	Pearson r = 0.56	0.56	0.633	450
	453	Increasing social job resources	Pearson r = 0.23	0.23	0.234	450
	453	Increasing challenging job demands	Pearson r = 0.47	0.47	0.510	450
	453	Decreasing hindering job demands	Pearson r = −0.01	−0.01	−0.010	450
De Beer et al., 2016, mining sample	260	Integrated crafting of structural resources and challenging job demands	SEM β = 0.33	0.33	0.343	257
	260	Increasing social job resources	SEM β = 0.33	0.33	0.343	257
De Beer et al., 2016, manufacturing sample	210	Integrated crafting of structural resources and challenging job demands	SEM β = 0.56	0.56	0.633	207
	210	Increasing social job resources	SEM β = 0.24	0.24	0.245	207
Petrou et al., 2018	368	Seeking resources	LCS γ = 0.17	0.17	0.172	365
	368	Reducing demands	LCS γ = −0.14	−0.14	−0.141	365
Muttar et al., 2024	276	Increasing structural job resources	Standardized β = 0.22	0.22	0.23	273
	276	Decreasing hindering job demands	Standardized β = 0.35	0.35	0.36	273
	276	Increasing social job resources	Standardized β = 0.30	0.30	0.31	273
	276	Increasing challenging job demands	Standardized β = 0.12	0.12	0.12	273
Sakuraya et al., 2017	894	Increasing structural job resources	Pearson r = 0.50	0.50	0.55	891
	894	Increasing social job resources	Pearson r = 0.30	0.30	0.31	891
	894	Increasing challenging job demands	Pearson r = 0.54	0.54	0.60	891
	894	Decreasing hindering job demands	Pearson r = 0.00	0.00	0.00	891
Moulik and Giri, 2024	310	Increasing structural job resources	Pearson r = 0.60	0.60	0.693	307
	310	Increasing social job resources	Pearson r = 0.68	0.68	0.83	307
	310	Increasing challenging job demands	Pearson r = 0.67	0.67	0.81	307
Ariani, 2023	1,032	Seeking job resources	Pearson r = 0.32	0.32	0.34	1,029
	1,032	Seeking job challenges	Pearson r = 0.47	0.47	0.51	1,029
	1,032	Reducing job demands	Pearson r = 0.23	0.23	0.24	1,029
Chen et al., 2024	733	Seeking job resources	Pearson r = 0.44	0.44	0.47	730
	733	Seeking job challenges	Pearson r = 0.57	0.57	0.65	730
	733	Reducing job demands	Pearson r = 0.45	0.45	0.48	730
Wang et al., 2024	308	Seeking job resources	Pearson r = 0.55	0.55	0.62	305
	308	Seeking job challenges	Pearson r = 0.53	0.53	0.59	305
	308	Reducing job demands	Pearson r = −0.22	−0.22	−0.22	305

Effect estimates are reported using the original job crafting dimension labels provided in each study. Only estimates that could be mapped with sufficient conceptual comparability to one of the four focal JD–R dimensions were included in the corresponding pooled models; broader or integrated constructs were retained in the table for transparency but were not assigned *post hoc* to a single dimension. SEM, structural equation modeling; LCS, latent change score.

### 2.5 Risk of bias assessment

Risk of bias was assessed using a design-specific JBI framework. Given the methodological heterogeneity of the included evidence, a single appraisal tool was not imposed across all studies. Instead, each study was evaluated using the Joanna Briggs Institute critical appraisal checklist matched to its design, including the Analytical Cross-Sectional Checklist, Cohort Checklist, and Quasi-Experimental Checklist, as appropriate (Aromataris et al., 2024; Barker et al., 2024, 2025, 2026). This design-sensitive approach was adopted to ensure that the appraisal remained anchored in the principal threats to validity associated with each study type rather than applying a uniform framework that might inadequately capture design-specific sources of bias.

Risk-of-bias judgments were made independently by two reviewers. To support transparent comparison across heterogeneous study designs, the original design-specific appraisals were subsequently harmonized into five review-level domains: participant selection, measurement bias, confounding and temporality, missing data, and reporting bias. Importantly, these harmonized domains were used as a comparative framework for cross-study synthesis only and did not replace the original design-specific judgments on which the primary appraisal was based.

For each harmonized domain, judgments were assigned as Low, Moderate, or High risk of bias. Low risk was assigned when the relevant methodological issue appeared to have been addressed appropriately and described with sufficient clarity. Moderate risk was assigned when some limitations were evident but were not considered severe enough to substantially compromise interpretability. High risk was assigned when a substantial threat to internal validity was present, including serious concerns related to sampling procedures, measurement quality, temporal ordering, control of confounding, incomplete outcome information, or reporting transparency. Particular interpretive weight was given to confounding and temporality because a substantial proportion of the included evidence was cross-sectional and therefore inherently limited with respect to directional inference.

Inter-rater agreement for the harmonized appraisal procedure was quantified using Cohen’s kappa. Overall agreement across the five harmonized domains was substantial (κ = 0.715), supporting the reliability of the review-level coding procedure. Overall study-level risk was determined qualitatively from the pattern of domain-level judgments rather than by numerical score summation. This decision was made because simple additive scoring may obscure the relative importance of different sources of bias and is not fully consistent with the logic of design-specific critical appraisal. For transparent reporting, the harmonized judgments were summarized in tabular form and visualized using traffic-light and summary plots.

### 2.6 Meta-analytic technique and statistics

All primary meta-analytic computations were conducted in R (v4.4.1), whereas the final forest plots were produced in Stata (v18.0) for graphical presentation (Fisher et al., 2022; Viechtbauer, 2010). Following effect-size extraction and standardization, all coefficients were analyzed in the Fisher’s *z* metric to stabilize sampling variance and improve distributional normality. Pooled estimates and confidence intervals were subsequently transformed back into Pearson’s r for interpretation, and all analyses were weighted by the inverse of the sampling variance.

Given the methodological and operational heterogeneity of the included studies, the primary synthesis was based on random-effects models using the restricted maximum-likelihood (REML) estimator (Veroniki et al., 2016). Separate meta-analytic models were fitted for increasing structural job resources, increasing social job resources, increasing challenging job demands, and decreasing hindering job demands in order to preserve conceptual differentiation within the JD–R framework. Heterogeneity was evaluated using Cochran’s Q, I^2^, and τ^2^. When studies reported conceptually adjacent labels, such as seeking resources, seeking challenges, or reducing demands, these estimates were coded into the closest corresponding JD–R-based dimension when conceptual comparability was judged sufficient (Cochran, 1954; Higgins and Thompson, 2002).

The primary quantitative synthesis was intentionally inclusive with respect to conceptually comparable and statistically convertible estimates, because the available literature was limited and diverse in both design and reporting practice. At the same time, this inclusiveness was bounded by explicit comparability rules: studies were entered only when the focal estimate could be aligned with a JD–R-based crafting dimension and interpreted as a standardized association with work engagement in the direction relevant to the review question. Studies with non-comparable analytic direction, highly aggregated reporting, or insufficiently aligned operationalizations were retained for narrative consideration but excluded from pooled estimation.

To evaluate the robustness of the primary findings, several sensitivity analyses were conducted. First, leave-one-out analyses were used to determine whether any single study or analytically distinct sample exerted disproportionate influence on the pooled estimates. Second, design-based sensitivity analyses excluded cross-sectional and dyadic cross-sectional evidence where feasible, thereby restricting the synthesis to studies with comparatively stronger temporal structure. Third, operationalization-based sensitivity analyses examined whether the pooled estimates were materially altered when broader, partially integrated, or otherwise less directly aligned operationalizations of the focal dimensions were excluded. These analyses were intended not as alternative primary models, but as boundary tests of the stability and interpretability of the main findings.

Given the limited number of studies contributing to the dimension-specific syntheses, formal tests of funnel-plot asymmetry and small-study effects were not conducted because their statistical power and interpretability would be limited, particularly in the presence of substantial between-study heterogeneity. Publication bias and small-study effects were therefore considered as potential limitations when interpreting the pooled estimates.

For visual presentation, dimension-specific forest plots were generated in Stata using the Fisher’s z effect sizes and corresponding standard errors derived from the R workflow. Each plot displayed the individual study estimates, their 95% confidence intervals, and the pooled random-effects REML estimate (Veroniki et al., 2016). Study markers were scaled according to inverse-variance weight, and heterogeneity statistics were reported directly in the plots.

## 3 Results

### 3.1 Risk of bias across included studies

Given the methodological heterogeneity of the 15 studies included in the quantitative synthesis, risk-of-bias judgments were first derived using the JBI checklist corresponding to each study design and were subsequently harmonized into common review-level domains. This two-stage procedure was considered necessary because the evidence base comprised cross-sectional surveys, longitudinal studies, diary-based repeated-measures designs, dyadic studies, and quasi-experimental evidence, each of which is associated with partly distinct threats to internal validity. The resulting harmonized judgments are presented in Table 3 and summarized visually in Figure 2.

**TABLE 3:** Harmonized risk-of-bias judgments based on a design-specific JBI approach.

References	Design	JBI appraisal tool	Participant selection	Measurement bias	Confounding and temporality	Missing data	Reporting bias	Overall judgment
Bakker et al., 2016	Dyadic cross-sectional survey	JBI Analytical Cross-Sectional Checklist	Moderate	Low	High	Moderate	Low	High
Tims et al., 2015	Three-wave longitudinal study	JBI Cohort Checklist	Moderate	Low	Moderate	Moderate	Low	Moderate
Petrou et al., 2012	Five-day diary study	JBI Cohort Checklist	Moderate	Low	Moderate	Moderate	Low	Moderate
Dubbelt et al., 2019	Longitudinal and quasi-experimental evidence	JBI Cohort Checklist/JBI Quasi-Experimental Checklist	High	Moderate	High	Moderate	Low	High
Tims et al., 2013	Three-wave longitudinal study	JBI Cohort Checklist	Moderate	Moderate	Moderate	Moderate	Low	Moderate
Bakker et al., 2012	Dyadic cross-sectional survey	JBI Analytical Cross-Sectional Checklist	Moderate	Low	High	Moderate	Low	High
Moreira et al., 2022	Cross-sectional survey	JBI Analytical Cross-Sectional Checklist	Moderate	Low	High	Moderate	Low	High
De Beer et al., 2016	Cross-sectional multi-group survey	JBI Analytical Cross-Sectional Checklist	Moderate	Moderate	High	Moderate	Low	High
Petrou et al., 2018	Three-wave longitudinal study with latent change score analysis	JBI Cohort Checklist	Moderate	Low	Moderate	Moderate	Low	Moderate
Muttar et al., 2024	Cross-sectional survey	JBI Analytical Cross-Sectional Checklist	Moderate	Moderate	High	Low	Low	High
Sakuraya et al., 2017	Cross-sectional internet survey	JBI Analytical Cross-Sectional Checklist	Moderate	Low	High	Low	Low	Moderate
Moulik and Giri, 2024	Cross-sectional survey	JBI Analytical Cross-Sectional Checklist	Moderate	Low	High	Moderate	Low	Moderate
Ariani, 2023	Cross-sectional survey	JBI Analytical Cross-Sectional Checklist	Moderate	Moderate	High	Moderate	Low	Moderate
Chen et al., 2024	Cross-sectional survey	JBI Analytical Cross-Sectional Checklist	Moderate	Moderate	High	High	Low	High
Wang et al., 2024	Three-wave longitudinal study	JBI Cohort Checklist	Moderate	Low	Moderate	Moderate	Low	Moderate

Cross-sectional studies were appraised using the JBI Critical Appraisal Checklist for Analytical Cross-Sectional Studies, longitudinal and diary-based studies using the JBI Critical Appraisal Checklist for Cohort Studies, and quasi-experimental evidence using the JBI Critical Appraisal Checklist for Quasi-Experimental Studies. To facilitate comparison across heterogeneous study designs, judgments were harmonized into five common domains: participant selection, measurement bias, confounding and temporality, missing data, and reporting bias. Overall judgments were determined qualitatively from the pattern of domain-level concerns rather than by summing checklist scores.

**FIGURE 2:**
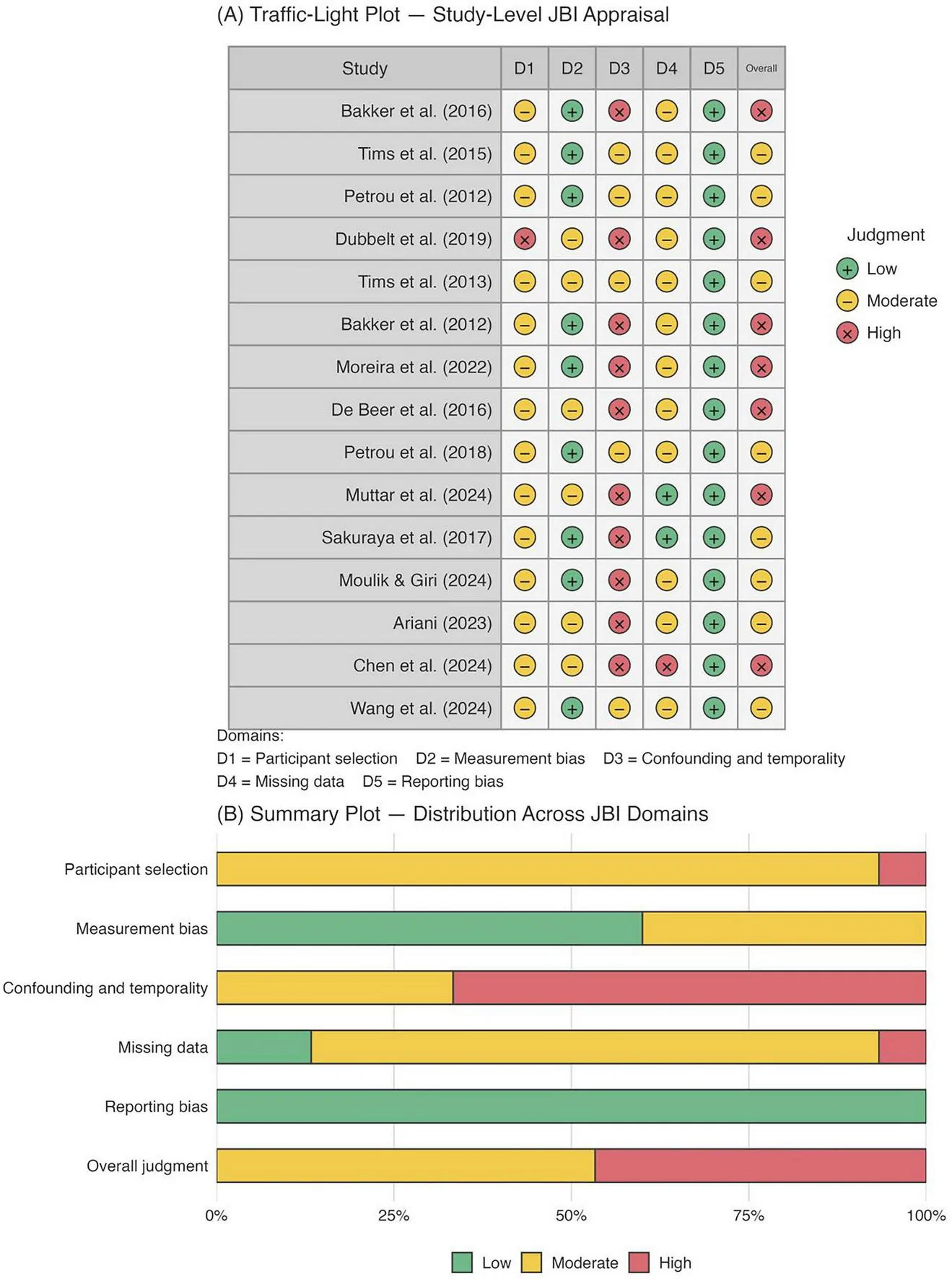
Harmonized risk-of-bias judgments across studies included in the quantitative synthesis. **(A)** Presents study-level judgments across five harmonized domains and the overall judgment. **(B)** Shows the distribution of Low, Moderate, and High judgments across the 15 included studies. D1, participant selection; D2, measurement bias; D3, confounding and temporality; D4, missing data; D5, reporting bias.

Across the included studies, the most consistent source of concern was confounding and temporality. This pattern was especially pronounced in the cross-sectional evidence, where job crafting and work engagement were typically assessed concurrently, thereby limiting confidence in directional interpretation and making residual confounding difficult to exclude. By contrast, the longitudinal and diary-based studies generally provided a stronger basis for temporal interpretation, although these designs were not free from limitation, particularly where attrition, incomplete follow-up information, or analytically complex within-person structures constrained causal inference. Participant selection was most often judged as Moderate, reflecting recurring concerns about convenience-based organizational samples, restricted occupational groups, and the limited representativeness typical of single-sector or internet-based recruitment strategies. Missing data was also most commonly rated as Moderate, although a small number of studies were judged more favorably when reporting and handling procedures appeared more transparent.

In contrast, measurement bias and reporting bias were generally less problematic across the evidence base. Most studies relied on established operationalizations of job crafting and work engagement, commonly derived from the JD–R tradition and the Utrecht Work Engagement Scale or closely related measures, which supported relatively stronger judgments in the measurement domain. Reporting bias was likewise more often judged as Low, suggesting that the main limitations of the included studies lay less in selective reporting or instrument choice than in design-related constraints on temporal ordering and confound control. Taken together, these patterns indicate that the evidence base was characterized predominantly by Moderate to High risk of bias, with the principal threats arising from confounding, temporal ambiguity, and, in some cases, participant selection. Accordingly, although the included studies were considered sufficiently rigorous to support quantitative synthesis, the pooled estimates should be interpreted primarily as associational rather than strongly causal in nature.

As shown in Figure 2, risk-of-bias judgments were not evenly distributed across domains. High-risk judgments clustered primarily in confounding and temporality, whereas Low-risk judgments were more common in reporting bias and, to a lesser extent, measurement bias. Selection-related concerns were more mixed but tended to fall in the Moderate range. This overall configuration suggests that the principal inferential limitation of the literature lies not in how job crafting and work engagement were measured, but in the predominance of observational designs that constrain temporal and causal interpretation.

### 3.2 Dimension-specific associations between job crafting and work engagement

To clarify whether different job crafting dimensions showed comparable or differentiated relationships with work engagement, separate random-effects meta-analytic models were estimated for each of the four focal JD–R-based dimensions. Figure 3 presents these dimension-specific syntheses in a single four-panel display: increasing structural job resources (Figure 3A), increasing social job resources (Figure 3B), increasing challenging job demands (Figure 3C), and decreasing hindering job demands (Figure 3D). The panels are combined for visual presentation only; each pooled estimate was obtained from a separate dimension-specific meta-analytic model.

**FIGURE 3:**
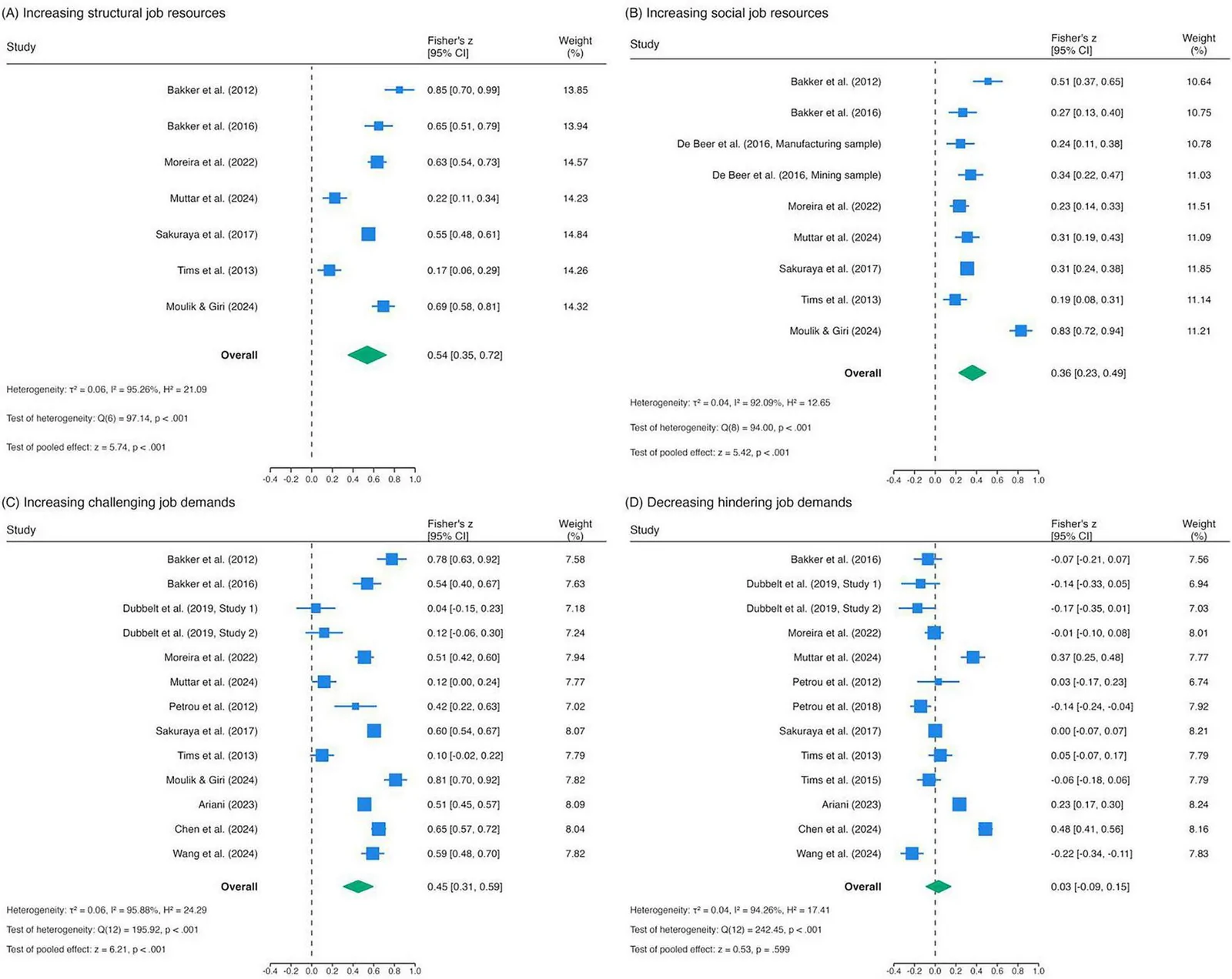
Dimension-specific meta-analytic associations between job crafting and work engagement. **(A)** Presents increasing structural job resources, **(B)** increasing social job resources, **(C)** increasing challenging job demands, and **(D)** decreasing hindering job demands. Squares represent individual study estimates, with marker size proportional to random-effects inverse-variance weight; horizontal lines indicate 95% confidence intervals. Diamonds represent pooled random-effects REML estimates. Effect sizes are displayed on the Fisher’s *z* scale.

For increasing structural job resources, seven effect estimates were synthesized (Bakker et al., 2012, 2016; Moreira et al., 2022; Muttar et al., 2024; Sakuraya et al., 2017; Tims et al., 2013; Moulik and Giri, 2024). The random-effects REML model yielded a significant positive pooled association with work engagement (Figure 3A; Fisher’s *z* = 0.54, 95% CI [0.35, 0.72], z = 5.74, *p* < 0.001). Substantial between-study heterogeneity was observed [τ^2^ = 0.06, I^2^ = 95.26%, Q_(6)_ = 97.14, *p* < 0.001], indicating considerable variation in the magnitude of the association across studies.

For increasing social job resources, nine effect estimates were included (Bakker et al., 2012, 2016; De Beer et al., 2016, manufacturing sample; De Beer et al., 2016, mining sample; Moreira et al., 2022; Muttar et al., 2024; Sakuraya et al., 2017; Tims et al., 2013; Moulik and Giri, 2024). The pooled association with work engagement was positive and statistically significant (Figure 3B; Fisher’s *z* = 0.36, 95% CI [0.23, 0.49], z = 5.42, p < 0.001). Heterogeneity was also substantial [τ^2^ = 0.04, I^2^ = 92.09%, Q_(8)_ = 94.00, *p* < 0.001], indicating marked variability across the contributing estimates.

For increasing challenging job demands, 13 effect estimates were synthesized (Bakker et al., 2012, 2016; Dubbelt et al., 2019, Study 1; Dubbelt et al., 2019, Study 2; Moreira et al., 2022; Muttar et al., 2024; Petrou et al., 2012; Sakuraya et al., 2017; Tims et al., 2013; Moulik and Giri, 2024; Ariani, 2023; Chen et al., 2024; Wang et al., 2024). The random-effects model indicated a significant positive pooled association with work engagement (Figure 3C; Fisher’s *z* = 0.45, 95% CI [0.31, 0.59], z = 6.21, *p* < 0.001). The estimates were highly heterogeneous [τ^2^ = 0.06, I^2^ = 95.88%, Q_(12)_ = 195.92, *p* < 0.001], suggesting substantial variation in association magnitude across studies.

For decreasing hindering job demands, 13 effect estimates were included (Bakker et al., 2016; Dubbelt et al., 2019, Study 1; Dubbelt et al., 2019, Study 2; Moreira et al., 2022; Muttar et al., 2024; Petrou et al., 2012, 2018; Sakuraya et al., 2017; Tims et al., 2015; Ariani, 2023; Chen et al., 2024; Wang et al., 2024). The pooled association with work engagement remained small and statistically non-significant (Figure 3D; Fisher’s *z* = 0.03, 95% CI [−0.09, 0.15], z = 0.53, *p* = 0.599). Substantial heterogeneity was nevertheless present [τ^2^ = 0.04, I^2^ = 94.26%, Q_(12)_ = 242.45, *p* < 0.001], indicating considerable variability among the individual study estimates despite the near-null pooled association.

Across the four dimension-specific models, the pattern of associations with work engagement was clearly differentiated. Figure 4 brings the pooled estimates onto a common Pearson’s r scale to support direct descriptive comparison across dimensions, together with their 95% confidence intervals. The figure is intended as a visual summary of the overall pattern and should not be interpreted as a formal test of between-dimension differences.

**FIGURE 4:**
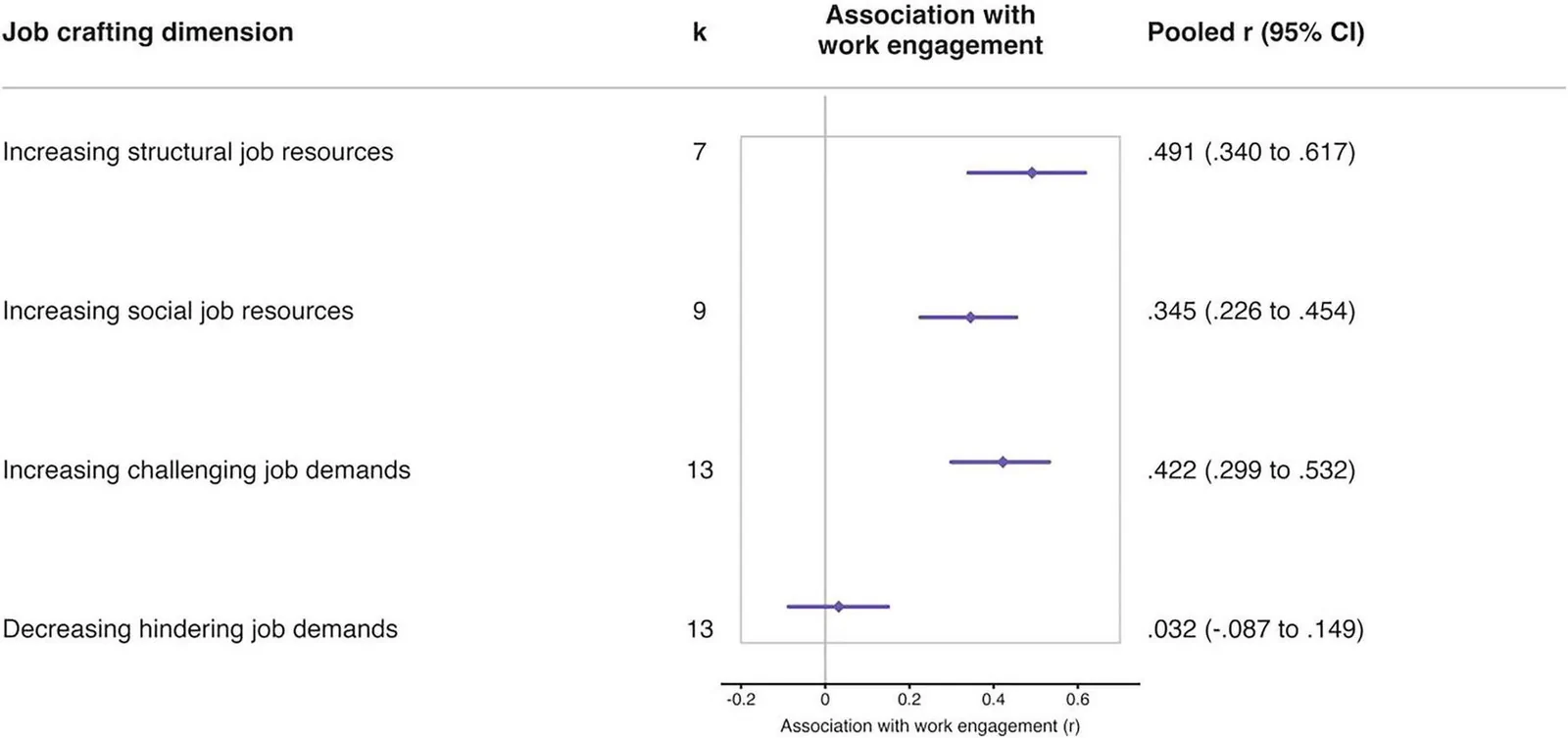
Summary forest plot of dimension-specific pooled associations between job crafting and work engagement. Points represent random-effects pooled Pearson’s r estimates, and horizontal lines indicate 95% confidence intervals. Estimates were obtained from separate dimension-specific meta-analytic models and are displayed together for descriptive comparison. k denotes the number of effect estimates contributing to each dimension-specific synthesis. The figure does not represent a formal statistical test of between-dimension differences.

### 3.3 Sensitivity analyses

#### 3.3.1 Leave-one-out analyses

Leave-one-out analyses were conducted separately for each job crafting dimension to examine whether the pooled estimates or heterogeneity statistics were disproportionately influenced by any single study or analytically distinct sample. For increasing structural job resources, the association with work engagement remained positive and statistically significant across all seven re-estimated models, with Fisher’s *z* ranging from 0.487 to 0.597 and all 95% confidence intervals remaining above zero. The smallest pooled estimate followed omission of Bakker et al. (2012), whereas the largest followed omission of Tims et al. (2013). Heterogeneity changed only modestly relative to the primary model (I^2^ = 95.26%), ranging from 93.22% after omission of Tims et al. (2013) to 96.15% after omission of Bakker et al. (2016). The persistence of both the positive association and high heterogeneity indicates that neither was attributable to a single study.

For increasing social job resources, Fisher’s *z* ranged from 0.296 to 0.380 across the nine leave-one-out models, and the association remained statistically significant in every iteration. In contrast to the structural-resources model, heterogeneity was more sensitive to study omission. Removing Moulik and Giri (2024) reduced I^2^ from 92.09% in the primary model to 54.15%, while the pooled estimate remained positive and statistically significant (Fisher’s *z* = 0.296, 95% CI [0.238, 0.354], *p* < 0.001). Omission of any other estimate left I^2^ above 91%. Thus, Moulik and Giri (2024) contributed substantially to between-study heterogeneity, but the positive pooled association did not depend on its inclusion.

For increasing challenging job demands, the leave-one-out estimates were similarly stable. Fisher’s *z* varied from 0.420 to 0.483 across 13 iterations, with every 95% confidence interval excluding zero. The lowest estimate was obtained after omission of Moulik and Giri (2024), whereas omission of Dubbelt et al. (2019), Study 1) produced the highest. Heterogeneity remained very high in all specifications, ranging from I^2^ = 95.46% to 96.44%, compared with 95.88% in the primary model. These limited changes indicate that the positive association and substantial between-study variability were distributed across the evidence base rather than driven by an individual estimate.

For decreasing hindering job demands, pooled Fisher’s *z* remained close to zero across all 13 iterations, ranging from −0.007 to 0.054, and every 95% confidence interval included zero. The largest reduction in heterogeneity occurred after omission of Chen et al. (2024), when I^2^ declined from 94.26% to 90.02%; the corresponding pooled estimate nevertheless remained essentially null (Fisher’s z = −0.007, 95% CI [−0.105, 0.092], *p* = 0.894). Other omissions produced only limited changes in the pooled estimate and heterogeneity. The leave-one-out analyses therefore did not alter the substantive interpretation of any dimension: the associations for structural resources, social resources, and challenging demands remained positive and statistically significant, whereas decreasing hindering job demands remained weak and non-significant. Detailed results of the leave-one-out analyses are reported in Supplementary Tables 1–4.

#### 3.3.2 Design-based sensitivity analyses

Design-based sensitivity analyses examined whether the primary estimates were materially affected by the inclusion of cross-sectional evidence. Cross-sectional and dyadic cross-sectional studies were excluded, leaving only studies with stronger temporal structure. For increasing structural job resources and increasing social job resources, only one eligible non-cross-sectional estimate remained in each synthesis, and pooled re-estimation was therefore not considered sufficiently informative. Restricted models were estimated for increasing challenging job demands and decreasing hindering job demands.

For increasing challenging job demands, restriction to five non-cross-sectional estimates attenuated the pooled association from r = 0.422 (95% CI [0.299, 0.532]) in the primary model to r = 0.252 (95% CI [0.043, 0.440], *p* = 0.019). The association nevertheless remained positive and statistically significant. Heterogeneity was lower than in the primary synthesis but remained substantial [Q_(4)_ = 49.44, *p* < 0.001, I^2^ = 89.95%, τ^2^ = 0.053], indicating that the positive association was not confined to cross-sectional studies, although its magnitude was reduced when the analysis was restricted to designs with clearer temporal ordering.

A different pattern emerged for decreasing hindering job demands. The primary model showed a small and non-significant association (r = 0.032, 95% CI [−0.087, 0.149], *p* = 0.599), whereas the seven-study non-cross-sectional model yielded a modest negative association (r = −0.096, 95% CI [−0.175, −0.017], *p* = 0.018). Heterogeneity also declined markedly, from I^2^ = 94.26% in the primary model to 58.05% in the restricted model [Q_(6)_ = 14.48, *p* = 0.025, τ^2^ = 0.006]. The shift in both the pooled estimate and heterogeneity suggests greater design sensitivity for decreasing hindering job demands than for increasing challenging job demands. Given the relatively small restricted evidence base, however, this result is better interpreted as evidence of design-related variation than as a replacement for the primary estimate. Detailed estimates from the design-based sensitivity analyses are reported in Supplementary Table 5.

#### 3.3.3 Operationalization-based sensitivity analyses

Operationalization-based sensitivity analyses examined whether the primary estimates were affected by the inclusion of broader or less directly aligned formulations of the four job crafting dimensions. The models were therefore re-estimated using only studies judged to provide more directly comparable operationalizations of the focal JD–R dimensions. For increasing structural job resources, all seven estimates met this criterion, leaving the restricted model identical to the primary synthesis (r = 0.491, 95% CI [0.340, 0.617], *p* < 0.001). For increasing social job resources, exclusion of the two De Beer et al. (2016) samples reduced the evidence base from 9 to 7 estimates, but the pooled association remained positive and statistically significant (r = 0.361, 95% CI [0.209, 0.495], *p* < 0.001). Heterogeneity remained high in the restricted model [Q_(6)_ = 91.62, *p* < 0.001, I^2^ = 94.16%, τ^2^ = 0.046].

For increasing challenging job demands, restricting the synthesis to seven more directly aligned estimates yielded a pooled association of r = 0.457 (95% CI [0.276, 0.607], *p* < 0.001), compared with r = 0.422 in the primary model. Between-study heterogeneity remained substantial [Q_(6)_ = 138.17, *p* < 0.001, I^2^ = 96.41%, τ^2^ = 0.077]. For decreasing hindering job demands, six estimates remained after the operationalization restriction. The pooled association remained small and non-significant (r = 0.045, 95% CI [−0.082, 0.171], *p* = 0.485), closely matching the near-null pattern of the primary model (r = 0.032). Although heterogeneity was reduced, it remained considerable [Q_(5)_ = 36.68, *p* < 0.001, I^2^ = 89.14%, τ^2^ = 0.022].

The operationalization-based analyses therefore preserved the main pattern of the primary findings. Positive associations remained evident for increasing structural job resources, increasing social job resources, and increasing challenging job demands, whereas decreasing hindering job demands continued to show a weak and non-significant association. At the same time, substantial residual heterogeneity remained in all four syntheses, indicating that differences in construct operationalization alone did not account for the observed between-study variability. The results of the leave-one-out, design-based, and operationalization-based sensitivity analyses are summarized together in Table 4. Detailed estimates from the operationalization-based sensitivity analyses are reported in Supplementary Table 6.

**TABLE 4:** Summary of sensitivity analyses across job crafting dimensions.

Dimension	Primary pooled r (95% CI)	Leave-one-out result	Design-restricted model	Operationalization-restricted model	Overall robustne
Increasing structural job resources	0.491 [0.340, 0.617]	Positive and significant in all iterations	Not pooled (k = 1)	k = 7, r = 0.491 [0.340, 0.617]	Robust
Increasing social job resources	0.345 [0.226, 0.454]	Positive and significant; heterogeneity fell markedly after omitting Moulik and Giri (2024)	Not pooled (k = 1)	k = 7, r = 0.361 [0.209, 0.495]	Robust, heterogeneity sensitive
Increasing challenging job demands	0.422 [0.299, 0.532]	Positive and significant in all iterations	k = 5, r = 0.252 [0.043, 0.440]	k = 7, r = 0.457 [0.276, 0.607]	Robust but attenuated by design restriction
Decreasing hindering job demands	0.032 [−0.087, 0.149]	Near-null and non-significant in all iterations	k = 7, r = −0.096 [−0.175, −0.017]	k = 6, r = 0.045 [−0.082, 0.171]	Design-sensitive

Primary estimates refer to the main dimension-specific random-effects models. Leave-one-out analyses sequentially omitted individual studies or analytically distinct samples. Design-restricted models excluded cross-sectional and dyadic cross-sectional evidence, whereas operationalization-restricted models retained only estimates with more directly comparable JD–R-congruent operationalizations. k denotes the number of effect estimates. Pooled associations are reported as Pearson’s r with 95% confidence intervals. Restricted design models were not pooled when fewer than three eligible estimates remained. Detailed results are provided in Supplementary Tables 1–6.

## 4 Discussion

### 4.1 Summary of findings

The present study examined whether the associations between work engagement and the four JD–R-based job crafting dimensions follow a similar pattern or differ in meaningful ways. The findings lend stronger support to the latter interpretation. Across the quantitative synthesis, the meta-analytic results showed that the direction, strength, and consistency of the associations varied across dimensions. Increasing structural job resources showed the strongest positive pooled association with work engagement, whereas increasing social job resources and increasing challenging job demands were also positively associated with work engagement, although to a more moderate extent. By contrast, decreasing hindering job demands displayed a near-null and generally non-significant pooled association. A similar pattern was evident in several of the individual studies included in the synthesis (e.g., Bakker et al., 2016; Tims et al., 2013; Petrou et al., 2012).

More specifically, increasing structural job resources showed the largest pooled association with work engagement (r = 0.491, 95% CI [0.340, 0.617]), followed by increasing challenging job demands (r = 0.422, 95% CI [0.299, 0.532]) and increasing social job resources (r = 0.345, 95% CI [0.226, 0.454]). In contrast, decreasing hindering job demands showed a pooled association close to zero (r = 0.032, 95% CI [−0.087, 0.149]). These findings suggest that the four JD–R-based job crafting dimensions should not be assumed to be interchangeable with respect to their observed associations with work engagement. Rather, forms of job crafting oriented toward resource expansion or active engagement with challenge appear to show more consistent positive associations with work engagement than forms centered on reducing hindering demands.

This distinction is theoretically important because treating job crafting as a unitary or higher-order construct may obscure meaningful variation among its constituent dimensions. Our findings therefore support a dimension-specific interpretation of the relationship between job crafting and work engagement. They are broadly consistent with earlier meta-analytic evidence. Frederick and VanderWeele (2020), using repeated-measures designs, reported positive associations for structural, social, and challenging forms of job crafting and a weak or null association for decreasing hindering job demands. Rudolph et al. (2017) likewise identified positive associations between job crafting and work engagement, whereas Lichtenthaler and Fischbach (2019) distinguished between promotion- and prevention-focused forms of crafting. The current synthesis extends this literature by updating the evidence base, examining dimension-specific associations across multiple study designs, and evaluating their robustness through design- and operationalization-based sensitivity analyses. Nevertheless, several pooled estimates showed substantial between-study heterogeneity. The findings are therefore more informative about the overall pattern of associations across job crafting dimensions than about the precise magnitude of any individual pooled estimate.

### 4.2 Theoretical interpretation of the differential associations within the JD–R framework

The differentiated pattern observed in the present meta-analysis is broadly consistent with the theoretical logic of the JD–R framework (Bakker and Demerouti, 2007). Within this perspective, work engagement is primarily linked to motivational processes supported by the availability, acquisition, and activation of job resources, as well as by the pursuit of challenging demands that may foster learning, mastery, and involvement in work. Against this background, it is not surprising that increasing structural job resources, increasing social job resources, and increasing challenging job demands all showed positive associations with work engagement, even if the strength of these associations was not identical across dimensions. These forms of job crafting appear to align more closely with the motivational pathway emphasized in the JD–R model, insofar as they involve employees actively expanding the conditions that are commonly associated with energy, dedication, and task involvement.

The particularly strong association observed for increasing structural job resources may be understood in light of the central role that autonomy, opportunities for development, and skill-related resources occupy within the JD–R tradition (Lee and Jo, 2023). Structural resources may be especially relevant because they are relatively proximal to employees’ sense of competence and control over their work, both of which have been consistently linked to engagement-related experiences (Shuck and Herd, 2012; Hakanen et al., 2024). Increasing social job resources also showed a positive association, although a comparatively smaller one. One possible interpretation is that social resources such as feedback, advice, and supervisory support are undoubtedly valuable, yet their relationship with engagement may depend more strongly on contextual conditions, including leadership climate, interpersonal norms, and the degree to which such resources can be mobilized effectively in a given setting (Calluso, 2025). In this respect, the present findings suggest that not all forms of resource-seeking are associated with work engagement to the same extent, even when they are grouped together within the broader domain of job resources.

A similar logic applies to increasing challenging job demands. Within the JD–R framework, challenging demands are often distinguished from hindering demands because they may be experienced not only as effortful but also as meaningful, growth-relevant, or competence-enhancing (Fernandez de Henestrosa et al., 2023; Demerouti and Bakker, 2011; Wang et al., 2021). When employees engage in forms of job crafting that involve seeking challenge, they may be orienting themselves toward more stimulating and developmentally relevant aspects of work. The positive association observed in the present synthesis is therefore compatible with the view that challenge-oriented crafting is more closely aligned with activation and involvement than with depletion (Li et al., 2022; Ortner et al., 2025). At the same time, the fact that this association was somewhat less robust than that observed for structural resources may indicate that challenging demands remain ambivalent in nature: although they may coincide with engagement under some conditions, they may also entail costs when opportunities for recovery or resource replenishment are limited.

By contrast, the weak and generally non-significant association observed for decreasing hindering job demands suggests that this dimension occupies a different theoretical position. Unlike the more expansion-oriented forms of job crafting, reducing hindering demands is less directly concerned with acquiring resources or approaching challenge and may instead reflect an effort to regulate strain, conserve energy, or minimize exposure to taxing aspects of work. From a JD–R perspective, such behavior may be more closely related to the management of overload or fatigue than to the motivational processes typically associated with work engagement (Li et al., 2025). This does not imply that decreasing hindering demands is maladaptive or unimportant. Rather, it suggests that its association with work engagement may be weaker, less direct, or more contingent on surrounding conditions than is the case for job crafting behaviors that expand resources or invite challenge.

More broadly, these findings contribute to ongoing theoretical clarification in the job crafting literature. A considerable portion of prior research has treated job crafting as a composite or higher-order construct, implicitly assuming that its constituent dimensions are functionally aligned. The present results do not support such an assumption in any simple sense. Instead, they suggest that the relationship between job crafting and work engagement is better understood at the level of specific behavioral dimensions, each of which may be associated with somewhat different psychological processes. In theoretical terms, the current findings are therefore more consistent with a differentiated account of job crafting within the JD–R framework than with a unitary conception in which all forms of crafting are expected to show similar associations with motivational outcomes (Jiang et al., 2022; Martínez-Díaz et al., 2023; Tims et al., 2013). In this sense, the contribution of the present study lies less in establishing a previously undocumented association than in updating and sharpening the evidence for dimension-specific patterning within the JD–R conceptualization of job crafting.

### 4.3 Limitations

Several limitations should be taken into account when interpreting the present findings. The quantitative evidence base remained relatively modest. Although 25 studies were retained for the narrative synthesis, 15 entered the quantitative synthesis, and the number of available effect sizes varied across dimensions. This constrained the precision of some pooled estimates and limited the extent to which more fine-grained supplementary analyses could be conducted, including the examination of a broader range of potential moderators.

A further limitation concerns the possibility of publication bias and small-study effects. The limited number of studies contributing to the dimension-specific syntheses reduces the reliability and interpretability of formal assessments of funnel-plot asymmetry. Consequently, the possibility that the pooled estimates were influenced by selective publication or systematically different estimates in smaller studies cannot be excluded. The pooled associations should therefore be interpreted alongside their confidence intervals, heterogeneity statistics, and sensitivity analyses rather than as definitive estimates of the underlying population associations.

The composition of the evidence base also imposes important constraints. A substantial proportion of the included studies was cross-sectional or dyadic cross-sectional, which restricts confidence in temporal ordering and leaves residual confounding difficult to exclude. This issue was already reflected in the design-sensitive appraisal framework adopted in the review, where particular weight was assigned to confounding and temporality because much of the available evidence was inherently limited with respect to directional inference. The design-based sensitivity analyses further underscored this point. After excluding cross-sectional evidence, pooled re-estimation remained feasible only for increasing challenging job demands and decreasing hindering job demands, whereas the evidence base for increasing structural job resources and increasing social job resources became too sparse to support stable restricted models. Accordingly, the present findings should not be interpreted as providing equally strong support across all dimensions with respect to temporal robustness.

Additional caution is warranted with regard to operationalization. Although the present review focused specifically on JD–R-based job crafting dimensions, the included studies did not operationalize these dimensions in fully identical ways. Several studies relied on conceptually adjacent labels such as seeking resources, seeking challenges, or reducing demands, whereas others used partially integrated formulations or broader measurement structures. This issue is particularly relevant in a literature where job crafting has often been conceptualized in broader or partially aggregated ways (e.g., Tims et al., 2012; Petrou et al., 2012; Lichtenthaler and Fischbach, 2019; Rudolph et al., 2017). These decisions were handled conservatively by coding only those estimates judged to be sufficiently comparable at the conceptual level. Even so, some degree of construct heterogeneity remained unavoidable, especially in light of prior indications that different job crafting forms may not be functionally interchangeable across outcomes (e.g., Petrou et al., 2012; Szőts-Kováts and Kiss, 2023).

The geographical distribution of the evidence base further narrows the scope of inference. Although the included studies were not confined to a single national context, the quantitative synthesis was disproportionately shaped by research conducted in Netherlands, with comparatively fewer studies drawn from Portugal, South Africa, Bahrain, Japan, and one multi-country European sample. This concentration is important because the expression and correlates of job crafting may vary across cultural and organizational contexts, including norms concerning autonomy, initiative, hierarchy, and social support at work. The present findings should therefore not be assumed to generalize equally across national settings, particularly in underrepresented regions.

Heterogeneity across studies should also be kept in view. Several of the primary syntheses were characterized by substantial between-study heterogeneity. Although leave-one-out and related sensitivity analyses indicated that the overall pattern of findings was broadly stable, the heterogeneity estimates suggest that the pooled estimates should not be interpreted as context-invariant associations. Rather, they reflect evidence drawn from studies that differed meaningfully in design, measurement format, analytic strategy, and contextual background. Accordingly, the present meta-analysis provides greater confidence in the overall pattern of associations across job crafting dimensions than in the exact magnitude of the corresponding pooled estimates.

These limitations do not negate the central contribution of the present meta-analysis, namely the clarification of a differentiated pattern of associations between specific job crafting dimensions and work engagement. They do, however, indicate that this contribution is better understood as evidence for relative dimensional patterning than as a set of equally precise and context-invariant estimates.

### 4.4 Practical implications and future research directions

From a practical perspective, the findings suggest that job crafting should not be treated as a functionally uniform strategy for promoting work engagement. Resource- and challenge-expanding forms of crafting showed consistently positive associations with engagement, whereas decreasing hindering job demands showed a near-null association in the primary synthesis. Organizations may therefore benefit from distinguishing among specific crafting behaviors when designing job crafting initiatives rather than encouraging job crafting as a broad and undifferentiated practice. This interpretation is broadly consistent with intervention research suggesting that structured job crafting programs can support more adaptive ways of shaping work when employees are encouraged to reflect on their work conditions and formulate concrete crafting goals (e.g., Costantini et al., 2022; van Wingerden and Poell, 2017).

The practical emphasis may differ across dimensions. Increasing structural job resources can be supported by providing employees with greater discretion over task methods, opportunities for skill development, and greater involvement in adapting workflows. Increasing social job resources may be facilitated through access to feedback, mentoring, peer consultation, and supervisor support. Challenge-oriented crafting may be encouraged through voluntary stretch assignments, developmental projects, or opportunities to assume meaningful additional responsibilities, provided that adequate resources and opportunities for recovery are available. By contrast, decreasing hindering job demands should not be assumed to function primarily as an engagement-enhancement strategy. Its primary pooled association with engagement was close to zero, while the design-restricted analysis indicated a modest negative association. Demand reduction may instead be more relevant to workload regulation, strain management, or recovery, depending on the organizational context. The dimension-specific findings and their practical interpretations are summarized in Table 5.

**TABLE 5:** Summary of dimension-specific associations with work engagement and practical interpretations.

Job crafting dimension	Pooled association with work engagement	Statistical pattern	Between-study heterogeneity	Practical interpretation
Increasing structural job resources	r = 0.491, 95% CI [0.340, 0.617]	Positive, statistically significant	I^2^ = 95.26%	Consider greater discretion over task methods, skill-development opportunities, and employee involvement in adapting workflows
Increasing social job resources	r = 0.345, 95% CI [0.226, 0.454]	Positive, statistically significant	I^2^ = 92.09%	Facilitate access to feedback, mentoring, peer consultation, and supervisor support
Increasing challenging job demands	r = 0.422, 95% CI [0.299, 0.532]	Positive, statistically significant	I^2^ = 95.88%	Consider voluntary stretch assignments, developmental projects, and meaningful additional responsibilities, accompanied by adequate resources and opportunities for recovery
Decreasing hindering job demands	r = 0.032, 95% CI [−0.087, 0.149]	Near-null, non-significant	I^2^ = 94.26%	Do not assume that reducing hindering demands will enhance engagement; it may be more relevant to workload regulation, strain management, or recovery

Pooled associations represent the primary random-effects estimates and are reported as Pearson’s r with 95% confidence intervals. Practical interpretations are evidence-informed implications rather than demonstrated causal effects of specific interventions. Pooled associations were estimated separately for each job crafting dimension and should not be interpreted as a formal statistical ranking of dimensions. Sensitivity analyses are summarized separately in Table 4.

These implications should nevertheless be interpreted cautiously. The pooled estimates represent associations rather than causal effects and do not establish the effectiveness of specific organizational interventions. Moreover, substantial between-study heterogeneity was observed for all four dimensions, indicating that the strength of these associations is likely to vary across samples and work settings. The sensitivity analyses also showed that some conclusions were more dependent on study design than others, particularly for decreasing hindering job demands. The present findings therefore provide a basis for differentiating and prioritizing forms of job crafting for further organizational consideration, rather than a prescriptive ranking of interventions.

Future research should place greater emphasis on longitudinal, diary, quasi-experimental, and intervention-based designs that permit stronger assessment of temporal ordering and within-person change. Greater consistency in construct operationalization is also needed. Studies would benefit from distinguishing clearly between the four JD–R-based dimensions and broader or partially aggregated formulations of job crafting, particularly when examining challenging and hindering demands. More standardized reporting of dimension-specific associations would also facilitate future quantitative synthesis and reduce uncertainty arising from differences in measurement and analytic specification.

Further work is needed to explain the substantial heterogeneity observed across the present syntheses. The evidence base remains geographically concentrated, with a notable proportion of studies conducted in Netherlands, limiting confidence in the generalizability of the pooled estimates across national and organizational contexts. Future studies should therefore examine whether the same dimension-specific pattern is observed across a broader range of countries, occupations, and industries, while testing contextual factors such as job autonomy, leadership support, organizational climate, workload, and occupational demands as potential moderators. Such work would help clarify not only whether particular forms of job crafting are associated with engagement, but also the conditions under which these associations are stronger or weaker.

## 5 Conclusion

The present meta-analysis indicates that job crafting dimensions conceptualized within the JD–R framework should not be treated as functionally equivalent when work engagement is the focal outcome. Resource- and challenge-oriented forms of job crafting showed more consistent positive associations with work engagement than forms centered on reducing hindering job demands. From a practical standpoint, this pattern supports a more differentiated approach in which structural-resource crafting, social-resource crafting, challenge-oriented crafting, and demand reduction are treated as distinct targets rather than as interchangeable components of a single intervention. However, because the present evidence is predominantly observational, these findings should inform the prioritization and design of organizational initiatives rather than be interpreted as evidence of their causal effectiveness.

Theoretically, the results are broadly consistent with the JD–R framework, insofar as job crafting dimensions more closely aligned with resource expansion and challenge engagement were also more consistently associated with work engagement, whereas demand-reducing forms appeared to occupy a less uniform position. The conclusions nevertheless warrant cautious interpretation in light of the modest evidence base, the predominance of observational designs, variation in operationalization, and the geographical concentration of studies in a limited number of national contexts. Future research should therefore test these patterns using stronger longitudinal and intervention-based designs, more conceptually precise operationalizations, and broader cultural and organizational coverage. Overall, the present study contributes most clearly by identifying a differentiated pattern of associations between job crafting dimensions and work engagement within the JD–R framework.

## Data Availability

The original contributions presented in this study are included in this article/Supplementary material, further inquiries can be directed to the corresponding author.
