# Supplementary material for: Differential associations between job crafting dimensions and work engagement: a meta-analytic review based on the JD–R framework
Source: Front Psychol. 2026 Oct 23;17:1856109. doi: 10.3389/fpsyg.2026.1856109 (PMC13739595; doi:10.3389/fpsyg.2026.1856109)
Supplement: Supplementary file 1 [file Table_1.DOCX]

**Supplementary Table S1. Leave-one-out analysis for increasing structural job resources**

This table summarizes the leave-one-out sensitivity analysis for increasing structural job resources, showing changes in the pooled effect and between-study heterogeneity after sequential omission of individual studies or analytically distinct samples.

| Omitted study | Pooled Fisher’s 𝑧 | 95% CI | p value | τ² | I² (%) |
| --- | --- | --- | --- | --- | --- |
| Primary model (no study omitted) | 0.537 | [0.354, 0.720] | < .001 | 0.06 | 95.26 |
| Bakker et al. (2012) | 0.487 | [0.305, 0.669] | < .001 | 0.05 | 94.79 |
| Bakker et al. (2016) | 0.519 | [0.307, 0.731] | < .001 | 0.07 | 96.15 |
| Moreira et al. (2022) | 0.521 | [0.306, 0.735] | < .001 | 0.07 | 95.68 |
| Muttar et al. (2024) | 0.589 | [0.409, 0.769] | < .001 | 0.05 | 94.41 |
| Sakuraya et al. (2017) | 0.535 | [0.317, 0.753] | < .001 | 0.07 | 95.17 |
| Tims et al. (2013) | 0.597 | [0.433, 0.761] | < .001 | 0.04 | 93.22 |
| Moulik and Giri (2024) | 0.511 | [0.303, 0.719] | < .001 | 0.06 | 95.76 |

**Note.** The primary model is shown in the first row for comparison. Each leave-one-out model was estimated using a random-effects REML approach after sequential omission of one study or analytically distinct sample. Effect sizes are reported in Fisher’s 𝑧 metric. τ² denotes the estimated between-study variance, and I² indicates the proportion of total variability attributable to between-study heterogeneity.

**Supplementary Table S2. Leave-one-out analysis for increasing social job resources**

This table summarizes the leave-one-out sensitivity analysis for increasing social job resources, showing changes in the pooled effect and between-study heterogeneity after sequential omission of individual studies or analytically distinct samples.

| Omitted study | Pooled Fisher’s 𝑧 | 95% CI | p value | τ² | I² (%) |
| --- | --- | --- | --- | --- | --- |
| Primary model (no study omitted) | 0.359 | [0.229, 0.489] | < .001 | 0.04 | 92.09 |
| Bakker et al. (2012) | 0.341 | [0.200, 0.482] | < .001 | 0.04 | 92.84 |
| Bakker et al. (2016) | 0.371 | [0.226, 0.515] | < .001 | 0.04 | 93.14 |
| De Beer et al. (2016, manufacturing sample) | 0.373 | [0.230, 0.517] | < .001 | 0.04 | 93.02 |
| De Beer et al. (2016, mining sample) | 0.362 | [0.214, 0.509] | < .001 | 0.04 | 93.21 |
| Moreira et al. (2022) | 0.376 | [0.232, 0.519] | < .001 | 0.04 | 92.27 |
| Muttar et al. (2024) | 0.366 | [0.219, 0.512] | < .001 | 0.04 | 93.11 |
| Sakuraya et al. (2017) | 0.366 | [0.219, 0.514] | < .001 | 0.04 | 91.73 |
| Tims et al. (2013) | 0.380 | [0.241, 0.520] | < .001 | 0.04 | 92.38 |
| Moulik and Giri (2024) | 0.296 | [0.238, 0.354] | < .001 | 0.00 | 54.15 |

**Note.** The primary model is shown in the first row for comparison. Each leave-one-out model was estimated using a random-effects REML approach after sequential omission of one study or analytically distinct sample. Effect sizes are reported in Fisher’s 𝑧 metric. τ² denotes the estimated between-study variance, and I² indicates the proportion of total variability attributable to between-study heterogeneity.

**Supplementary Table S3. Leave-one-out analysis for increasing challenging job demands**

This table summarizes the leave-one-out sensitivity analysis for increasing challenging job demands, showing changes in the pooled effect and between-study heterogeneity after sequential omission of individual studies or analytically distinct samples.

| Omitted study | Pooled Fisher’s 𝑧 | 95% CI | p value | τ² | I² (%) |
| --- | --- | --- | --- | --- | --- |
| Primary model (no study omitted) | 0.450 | [0.308, 0.593] | < .001 | 0.06 | 95.88 |
| Bakker et al. (2012) | 0.424 | [0.280, 0.568] | < .001 | 0.06 | 95.80 |
| Bakker et al. (2016) | 0.443 | [0.289, 0.597] | < .001 | 0.07 | 96.33 |
| Dubbelt et al. (2019, Study 1) | 0.483 | [0.345, 0.620] | < .001 | 0.06 | 95.52 |
| Dubbelt et al. (2019, Study 2) | 0.476 | [0.333, 0.620] | < .001 | 0.06 | 95.86 |
| Moreira et al. (2022) | 0.445 | [0.290, 0.600] | < .001 | 0.07 | 96.14 |
| Muttar et al. (2024) | 0.479 | [0.336, 0.621] | < .001 | 0.06 | 95.51 |
| Petrou et al. (2012) | 0.452 | [0.298, 0.606] | < .001 | 0.07 | 96.44 |
| Sakuraya et al. (2017) | 0.437 | [0.284, 0.589] | < .001 | 0.07 | 95.64 |
| Tims et al. (2013) | 0.481 | [0.340, 0.621] | < .001 | 0.06 | 95.49 |
| Moulik and Giri (2024) | 0.420 | [0.280, 0.561] | < .001 | 0.06 | 95.46 |
| Ariani (2023) | 0.445 | [0.290, 0.600] | < .001 | 0.07 | 95.66 |
| Chen et al. (2024) | 0.433 | [0.282, 0.584] | < .001 | 0.07 | 95.67 |
| Wang et al. (2024) | 0.438 | [0.285, 0.591] | < .001 | 0.07 | 96.18 |

**Note.** The primary model is shown in the first row for comparison. Each leave-one-out model was estimated using a random-effects REML approach after sequential omission of one study or analytically distinct sample. Effect sizes are reported in Fisher’s 𝑧 metric. τ² denotes the estimated between-study variance, and I² indicates the proportion of total variability attributable to between-study heterogeneity.

**Supplementary Table S4. Leave-one-out analysis for decreasing hindering job demands**

This table summarizes the leave-one-out sensitivity analysis for decreasing hindering job demands, showing changes in the pooled effect and between-study heterogeneity after sequential omission of individual studies or analytically distinct samples.

| Omitted study | Pooled Fisher’s 𝑧 | 95% CI | p value | τ² | I² (%) |
| --- | --- | --- | --- | --- | --- |
| Primary model (no study omitted) | 0.032 | [−0.087, 0.151] | .599 | 0.04 | 94.26 |
| Bakker et al. (2016) | 0.040 | [−0.087, 0.167] | .539 | 0.05 | 94.80 |
| Dubbelt et al. (2019, Study 1) | 0.045 | [−0.080, 0.169] | .483 | 0.04 | 94.73 |
| Dubbelt et al. (2019, Study 2) | 0.047 | [−0.076, 0.171] | .454 | 0.04 | 94.61 |
| Moreira et al. (2022) | 0.035 | [−0.094, 0.164] | .594 | 0.05 | 94.61 |
| Muttar et al. (2024) | 0.005 | [−0.111, 0.120] | .939 | 0.04 | 93.52 |
| Petrou et al. (2012) | 0.032 | [−0.096, 0.159] | .627 | 0.05 | 94.99 |
| Petrou et al. (2018) | 0.047 | [−0.079, 0.172] | .465 | 0.04 | 94.38 |
| Sakuraya et al. (2017) | 0.034 | [−0.095, 0.163] | .604 | 0.05 | 94.13 |
| Tims et al. (2013) | 0.030 | [−0.099, 0.159] | .650 | 0.05 | 94.82 |
| Tims et al. (2015) | 0.039 | [−0.089, 0.167] | .547 | 0.05 | 94.73 |
| Ariani (2023) | 0.014 | [−0.110, 0.138] | .829 | 0.04 | 93.46 |
| Chen et al. (2024) | −0.007 | [−0.105, 0.092] | .894 | 0.03 | 90.02 |
| Wang et al. (2024) | 0.054 | [−0.067, 0.174] | .380 | 0.04 | 94.01 |

**Note.** The primary model is shown in the first row for comparison. Each leave-one-out model was estimated using a random-effects REML approach after sequential omission of one study or analytically distinct sample. Effect sizes are reported in Fisher’s 𝑧 metric. τ² denotes the estimated between-study variance, and I² indicates the proportion of total variability attributable to between-study heterogeneity.

**Supplementary Table S5. Design-based sensitivity analyses of the pooled associations between job crafting dimensions and work engagement**

This table summarizes the design-based sensitivity analyses, comparing the primary models with models restricted to non-cross-sectional studies to examine whether the pooled associations and between-study heterogeneity were sensitive to study design.

| Job crafting dimension | Model | k | Pooled 𝑟 | 95% CI | p value (pooled effect) | Cochran’s Q (df) | I² (%) | τ² |
| --- | --- | --- | --- | --- | --- | --- | --- | --- |
| Increasing challenging job demands | Primary model | 13 | .422 | [.299, .532] | < .001 | 195.92 (12) | 95.88 | .060 |
| Increasing challenging job demands | Restricted to non-cross-sectional designs | 5 | .252 | [.043, .440] | .019 | 49.44 (4) | 89.95 | .053 |
| Decreasing hindering job demands | Primary model | 13 | .032 | [−.087, .149] | .599 | 242.45 (12) | 94.26 | .040 |
| Decreasing hindering job demands | Restricted to non-cross-sectional designs | 7 | −.096 | [−.175, −.017] | .018 | 14.48 (6) | 58.05 | .006 |

**Note.** Design-restricted models excluded cross-sectional and dyadic cross-sectional studies and retained only studies with comparatively stronger temporal structure. Restricted pooled models were not estimated for increasing structural job resources or increasing social job resources because only one eligible non-cross-sectional estimate remained for each dimension. k denotes the number of effect estimates included in each model. Pooled associations are reported as Pearson’s 𝑟 with 95% confidence intervals, although the meta-analytic models were estimated in Fisher’s 𝑧 metric using random-effects REML procedures. Q denotes Cochran’s heterogeneity statistic; I² represents the proportion of total variability attributable to between-study heterogeneity; and τ² denotes the estimated between-study variance.

**Supplementary Table S6. Operationalization-based sensitivity analyses across the four job crafting dimensions**

This table summarizes the operationalization-based sensitivity analyses, comparing the primary models with models restricted to estimates judged to provide more directly comparable JD–R-congruent operationalizations of each focal job crafting dimension.

| Job crafting dimension | Primary k | Restricted k | Restricted pooled 𝑟 | 95% CI | p value (pooled effect) | Cochran’s Q (df) | I² (%) | τ² |
| --- | --- | --- | --- | --- | --- | --- | --- | --- |
| Increasing structural job resources | 7 | 7 | .491 | [.340, .617] | < .001 | 97.14 (6) | 95.26 | .060 |
| Increasing social job resources | 9 | 7 | .361 | [.209, .495] | < .001 | 91.62 (6) | 94.16 | .046 |
| Increasing challenging job demands | 13 | 7 | .457 | [.276, .607] | < .001 | 138.17 (6) | 96.41 | .077 |
| Decreasing hindering job demands | 13 | 6 | .045 | [−.082, .171] | .485 | 36.68 (5) | 89.14 | .022 |

**Note.** “Primary k” refers to the number of effect estimates included in the main dimension-specific meta-analytic model, whereas “Restricted k” refers to the number retained after applying the operationalization-based restriction criterion. Restricted models included only estimates judged to provide more directly comparable JD–R-congruent operationalizations of the focal dimension. Pooled associations are reported as Pearson’s 𝑟 with 95% confidence intervals, although the models were estimated in Fisher’s 𝑧 metric using random-effects REML procedures. Q denotes Cochran’s heterogeneity statistic; I² represents the proportion of total variability attributable to between-study heterogeneity; and τ² denotes the estimated between-study variance.
